# Redefining governance: a critical analysis of sustainability transformation in e-governance

**DOI:** 10.3389/fdata.2024.1349116

**Published:** 2024-04-03

**Authors:** Qaiser Abbas, Tahir Alyas, Turki Alghamdi, Ahmad B. Alkhodre, Sami Albouq, Mushtaq Niazi, Nadia Tabassum

**Affiliations:** ^1^Faculty of Computer and Information Systems, Islamic University of Madinah, Madinah, Saudi Arabia; ^2^Department of Computer Science, Lahore Garrison University, Lahore, Pakistan; ^3^Department of Computer Science, Riphah International University, Sahiwal, Pakistan; ^4^Department of Computer Science, Virtual University of Pakistan, Lahore, Pakistan

**Keywords:** e-governance, cloud computing, big data, performance, secure data management, data modeling, sustainability

## Abstract

With the rapid growth of information and communication technologies, governments worldwide are embracing digital transformation to enhance service delivery and governance practices. In the rapidly evolving landscape of information technology (IT), secure data management stands as a cornerstone for organizations aiming to safeguard sensitive information. Robust data modeling techniques are pivotal in structuring and organizing data, ensuring its integrity, and facilitating efficient retrieval and analysis. As the world increasingly emphasizes sustainability, integrating eco-friendly practices into data management processes becomes imperative. This study focuses on the specific context of Pakistan and investigates the potential of cloud computing in advancing e-governance capabilities. Cloud computing offers scalability, cost efficiency, and enhanced data security, making it an ideal technology for digital transformation. Through an extensive literature review, analysis of case studies, and interviews with stakeholders, this research explores the current state of e-governance in Pakistan, identifies the challenges faced, and proposes a framework for leveraging cloud computing to overcome these challenges. The findings reveal that cloud computing can significantly enhance the accessibility, scalability, and cost-effectiveness of e-governance services, thereby improving citizen engagement and satisfaction. This study provides valuable insights for policymakers, government agencies, and researchers interested in the digital transformation of e-governance in Pakistan and offers a roadmap for leveraging cloud computing technologies in similar contexts. The findings contribute to the growing body of knowledge on e-governance and cloud computing, supporting the advancement of digital governance practices globally. This research identifies monitoring parameters necessary to establish a sustainable e-governance system incorporating big data and cloud computing. The proposed framework, Monitoring and Assessment System using Cloud (MASC), is validated through secondary data analysis and successfully fulfills the research objectives. By leveraging big data and cloud computing, governments can revolutionize their digital governance practices, driving transformative changes and enhancing efficiency and effectiveness in public administration.

## 1 Introduction

The advent of digital technology has revolutionized various aspects of governance, leading to the emergence of e-governance as a significant domain within public administration and policy. E-governance, characterized by integrating digital tools and processes in governmental operations, has been widely recognized for its potential to enhance efficiency, transparency, and citizen participation. However, as the global emphasis shifts toward sustainable development, it is imperative to critically analyze and redefine governance structures, particularly focusing on how e-governance can be harnessed to foster sustainability transformations.

This study endeavors to explore the intersection of e-governance and sustainability, providing a comprehensive overview of the current landscape and delineating the pathways through which digital governance can contribute to sustainable outcomes. It examines the role of e-governance in promoting environmental, social, and economic sustainability, thereby contributing to the achievement of the United Nations Sustainable Development Goals (SDGs). The analysis is anchored in a critical review of the literature and case studies that demonstrate innovative practices and challenges in integrating sustainability into e-governance.

The need for a sustainability-oriented redefinition of governance is underscored by the escalating environmental crises, growing social inequalities, and the urgent demand for inclusive economic development. As governments worldwide strive to meet these challenges, e-governance emerges as a pivotal tool in the transformation process. This study argues that while e-governance presents numerous opportunities for sustainability, it also entails complex challenges that require careful consideration and strategic planning.

**Figure 1:**
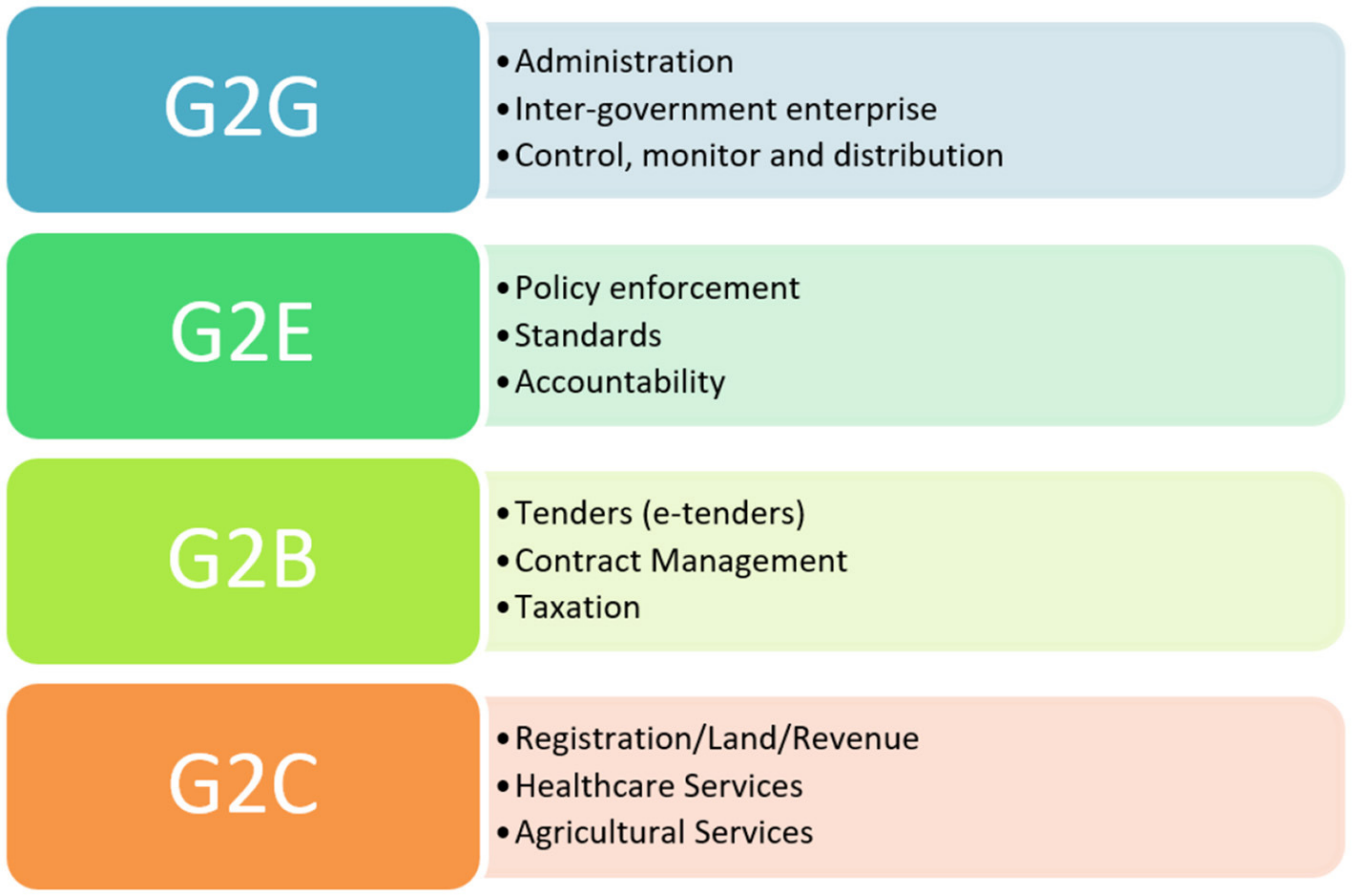
Types of e-governance applications.

This study aims to contribute to the discourse on sustainable development and digital governance through its critical analysis. It seeks to provide policymakers, practitioners, and scholars with insights into the effective integration of sustainability principles into e-governance strategies, thereby paving the way for a more resilient, equitable, and sustainable future. In doing so, it calls for a redefinition of governance that embraces technological innovation and prioritizes long-term sustainability as a core objective. There are four broad categories of applications if we consider e-governance over cloud computing, as shown in Figure 1. These four categories are defined based on the usage and relation of stakeholders; there are secondary and tertiary entities as well; however, here, only the main categories are mentioned to provide a comprehensive background on the concept of e-governance.

G2G (Government to Government) represents the interactions between different government agencies. G2E (Government to Employees) reflects the relationship between the government and its employees. G2B (Government to Business) indicates the interface between the government and businesses. G2C (Government to Citizens) represents the connection between the government and the general public.

It is recognized that the expansive nature of e-governance systems often results in the operation of numerous sub-systems in isolation, lacking an integrated monitoring mechanism. The absence of such a cohesive framework can lead to inefficiencies and hamper the sustainable evolution of e-governance. Cloud computing has emerged as a robust global platform that caters to diverse user needs, positioning it as an essential component for modern e-governance infrastructures. It is imperative, therefore, to investigate and establish a comprehensive framework that facilitates continuous monitoring. This framework would enable governments to make informed decisions about infrastructure modifications, platform upgrades, and the on-demand availability of Software as a Service (SaaS). Such a proactive monitoring system is not merely an enhancement to current e-governance practices but is a critical step toward strategic future-proofing and planning. This strategy would ensure that e-governance systems are not only efficient and responsive to current needs but are also sustainable and adaptable to the evolving technological landscape, underpinned by the principles of cloud computing.

This research delves into the intricate landscape of e-governance, seeking to thoroughly investigate its current condition. We aim to unravel the complexities and hurdles governments encounter as they attempt to deploy and integrate e-governance systems. In parallel, we explored the substantial advantages that could be harnessed by incorporating big data and cloud computing technologies into these systems. The study will provide a deep dive into the fundamental principles and innovative technologies that form the backbone of big data and cloud computing, assessing their potential application within the realm of e-governance.

Our inquiry extends to discern how the amalgamation of big data and cloud computing could revolutionize e-governance. We will focus on how these technologies can elevate data management and analytical capabilities, scale and adapt e-governance frameworks, and enhance the interaction and involvement of citizens. By exploring these technological synergies, the study seeks to illuminate pathways that lead to more dynamic, efficient, and participatory governance structures, aligning with the evolving digital landscape and the increasing demands of the public sector.

The transformation of e-governance through big data and cloud computing has the potential to bring significant benefits to society. These technologies can help to improve service delivery, increase the efficiency and effectiveness of government operations, and enhance citizen engagement and participation in governance. Growing importance of e-governance: In recent years, e-governance has become a crucial aspect of modern governance, as governments worldwide are leveraging technology to improve their services, increase transparency, and engage with citizens more effectively. Big data and cloud computing are two key technologies that have the potential to transform e-governance, making it more efficient, effective, and citizen-centric. Cloud computing has revolutionized how organizations store, process, and manage data. With the emergence of cloud-based services, governments can now leverage the power of cloud computing to deliver scalable, secure, and cost-effective e-governance solutions. Big data technologies can help governments store, process, and analyze large volumes of data in real time, enabling them to make more informed decisions and provide better services to citizens.

The emergence of cloud computing and its swift spread as a universal platform for individuals, corporations, and governments alike requires standardization for sustenance and growth. Specifically, in e-governance, cloud-based e-services need a monitoring framework that shall engage the parameters to highlight the strengths and weaknesses. Being a global system, the local culture, priorities, socio-economic politics, and many other factors are certain segments to be considered, but the global best practices and continuous development in e-governance can provide such parameters that are reliable and common in all regions. A monitoring framework for e-governance will improve the quality of public service and indicate new paths for social and technology research. E-governance is a worldwide phenomenon that is progressing with the emergence of new technologies. Providing services to a community is based on multiple local factors and parameters such as culture, priorities, and socio-economic values that lead to developing a complex, customized, and localized e-governance system. Cloud computing has emerged as a global platform for digital transformation and attracting e-governance systems with multi-fold benefits and facilities; therefore, it is required to identify the common parameters of e-governance systems to engage core-level standardization. The core parameters may lead toward identifying more common and standard sub-parameters to develop an e-governance system as a cloud service.

## 2 Literature review

The ability of individuals to effectively handle their finances plays a crucial role in determining the success of their entrepreneurial endeavors. This article utilizes panel data obtained from the China Household Finance Survey (C.H.F.S.) conducted in 2013, 2015, and 2017 to investigate the impact of financial capability on entrepreneurial performance in rural China. The findings reveal a positive correlation between financial capability and entrepreneurship's scale, profitability, and sustainability. Importantly, these results remain robust even when accounting for potential endogeneity. Moreover, it is observed that the effects of financial capability vary among different entrepreneurs (Yi et al., 2023).

In cloud computing, one cloud service provider with a certain number of services for end-use is a successful centralized setup that provides good performance. Countries worldwide have engaged in e-governance in some manner; developed countries have a more mature and comprehensive structure of applications and tools helping in e-services. Developing countries have deployed e-governance in a few critical domains and are struggling to extend the same services in other domains (Seifert, 2003; Almarabeh et al., 2016). The other impactful areas are transparency and reduction in corruption and corrupt practices. Deploying e-governance systems and sub-systems improves the productivity of the public sector, results in far better performance indicators, and a visibly different and significantly better response time on public queries and complaints (Zong and Wan, 2022). E-governance and the respective systems provide multifold benefits to the government, government officials, businesses, and citizens.

As a national hub, the e-governance system is the platform that engages the citizens to respond to their needs related to government departments, e.g., utility bills, tax returns, marriage, birth, and death data, while the other one is which citizens may hire in case of requirement such as medical emergency, police, fire brigade, emergency services, etc. The good practices in implementing the e-governance system and cultural acceptance are a segment on which governments must work more closely. One more factor is the financial impact; if the e-services are too expensive, then there are negligible chances for people to adapt (Seifert and Bonham, 2003).

The fundamental drivers of innovation lie within enterprises, universities, and scientific research institutions, collectively constituting the core subjects of innovation. The allocation and concentration of innovation resources wield a significant influence on innovation efficiency. This study investigates the innovation inequality among these three subjects (Industry—University—Research) in China. The analysis employs single-dimensional inequality synthesis, the multidimensional Gini coefficient, and the Atkinson–Kolm–Sen index to comprehensively assess and quantify innovation disparities (Xu et al., 2023). For governments, initially, e-governance has multiple costing factors, such as the development of e-governance tools, applications, and software. The engagement of cloud service providers bears a certain cost of cloud facilities that will cover the infrastructure, platforms, and software services (Seifert and Bonham, 2003). In the current era, software security (Liu et al., 2023a) is the function of application usage outside permission. This specific issue has always been a challenging environment where software engineers and developers have needed assistance and resources. User-side protection is changing, but it is still a problem due to the browsers' condition.

Effectively identifying and categorizing opinions in text using computational methods is crucial for enhancing understanding and providing improved services to online users in the digital environment. Despite its significance, achieving accurate and swift multi-label automatic classification remains a challenge. In this study, the focus is on refining the Multi-label K-Nearest Neighbors (MLkNN) classifier to address this limitation. The approach involves considering not only individual in-sentence features but also incorporating features from adjacent sentences and the entire text of the tweet. This adjustment enables iterative corrections in the multi-label emotion classification process, enhancing accuracy and efficiency (Liu et al., 2023a). The imperative for E-Government, or electronic government, is driven by the need for public administration to adapt and leverage digital technologies to enhance governance, service delivery, and citizen engagement (OECD, 2003). The structuring and planning phases of an e-governance system are comparatively easy. Still, the actual exercise, i.e., implementation of an e-governance system, is a challenge and requires a delicately designed strategy (Smith, 2002; Carvin et al., 2004).

The other dimension of initial costs includes the physical infrastructure to spread the Internet or cellular network with the provision to deliver a certain bandwidth for the end-users. Finally, training and educating relevant staff and citizens, as well as developing national-level programs, will be crucial for ensuring people understand how to use e-services, along with their prerequisites and benefits (Alshomrani and Qamar, 2013).

To fix this problem, continued research is being conducted by many organizations, like IBM, which has published the concept of the reverse proxy to increase security and authenticity. Similarly, large-scale corporate security demands and requirements are being satisfied using SSAL and other security tools by Force.com (Das et al., 2011).

As the Electronic-Based Government System evolves within the Department of Communication and Informatics of the Bandung City Government, developing an Enterprise Architecture (EA) becomes imperative to ensure the system's seamless integration and effective implementation. With a specific focus on the technology domain, this study adopts the TOGAF (The Open Group Architecture Framework) Architecture Development Method (ADM) to guide the analysis, design, and improvement of the technological aspects of the Electronic-Based Government System (Fahlevi and Nugraha, 2023).

The benefits of mobile governance are that it becomes more available, affordable, flexible, collaborative, and convenient because of mobility. Many states' governments are increasing internal competitiveness to improve the effectiveness of their programs externally. The rapidly growing usage of cellular mobile devices in terms of cell phones, tablets, and laptops, the possibilities to expand service quality, greater openness through transparent and user-friendly accessibility mechanisms for information, and new services that can engage with people all contribute to the case for mobile governance (Sharma and Thapliyal, 2011).

A secure platform is an integral element of digital governance, playing a crucial role in safeguarding the privacy, security, and reliability of electronic systems used to administer and provide public services. Interoperability and data exchange are imperative in digital governance, facilitating the seamless sharing of data, information, and resources among diverse government agencies and departments, irrespective of the platforms and technologies they employ (Malik et al., 2023).

Another advantage of using mobile e-governance and provisioning cloud services is that the solutions offered by the cloud service providers are less expensive than conventional Information Technology (IT) solutions. End-users always consider a few factors, such as there are no maintenance charges as the cloud service provider is responsible for service availability, and the end-user is free from looking into the resource machine maintenance and management issues, so organizations moving on the cloud are no longer investing in such parameters. The tools and facilities relevant to e-government represent the citizen priority dimension of any e-governance structure (Glick, 2009; Wyld, 2009).

With remote accessibility and e-governance applications, public officials can easily develop and monitor cases to be updated on current progress and use device features such as a geographical positioning system and camera to support such operations. An e-government scheme manages data related to cases securely and centrally. When caseworkers are more attentive and the evidence is correct, costs are minimized (Government of the United Kingdom, 2009; Tabassum et al., 2023). Citizens must have access to information to build a transparent government. Government can distribute information across multiple networks using geospatial technology and location data, which can be accessed at any time and from any location. Mobile technology expands the possibilities for political participation. The cloud technology in the backend provides all the utilities delivered by a mobile app. Cloud computing provides residents and government employees with high flexibility, scalability, and fast response times for mobile services^1^.

Text classification is recognized as a pivotal process for organizing online content to enhance communication in the digital media age. It involves establishing classification rules derived from text features, making the accuracy of feature selection foundational to the success of text classification. In the rapidly growing realm of Chinese electronic documents within the digital environment, scholars have developed numerous algorithms in recent years to refine feature selection for the automatic classification of Chinese texts (Liu et al., 2023b).

## 3 Proposed methodology

The “Transformation of Technological Advancement in Big Data and Cloud Computing for Digital E-Governance” conceptual solution involves leveraging big data and cloud computing technologies to improve e-governance systems, making them more efficient, effective, and citizen-centric. In this research, such parameters are critically important. This research aims to identify and develop a monitoring system to transform global e-governance into a standardized and systemic activity. The digital divide between developed and developing countries shall be reduced by adapting such frameworks and monitoring structures that lead to global standards. This research proposes a Monitoring and Assessment System using Cloud (MASC) for e-governance. MASC is focused on engaging global parameters to assess services and accordingly maintaining a database to use the system patterns for learning and strategy development for system sustainability and strategy alignment.

**Figure 2:**
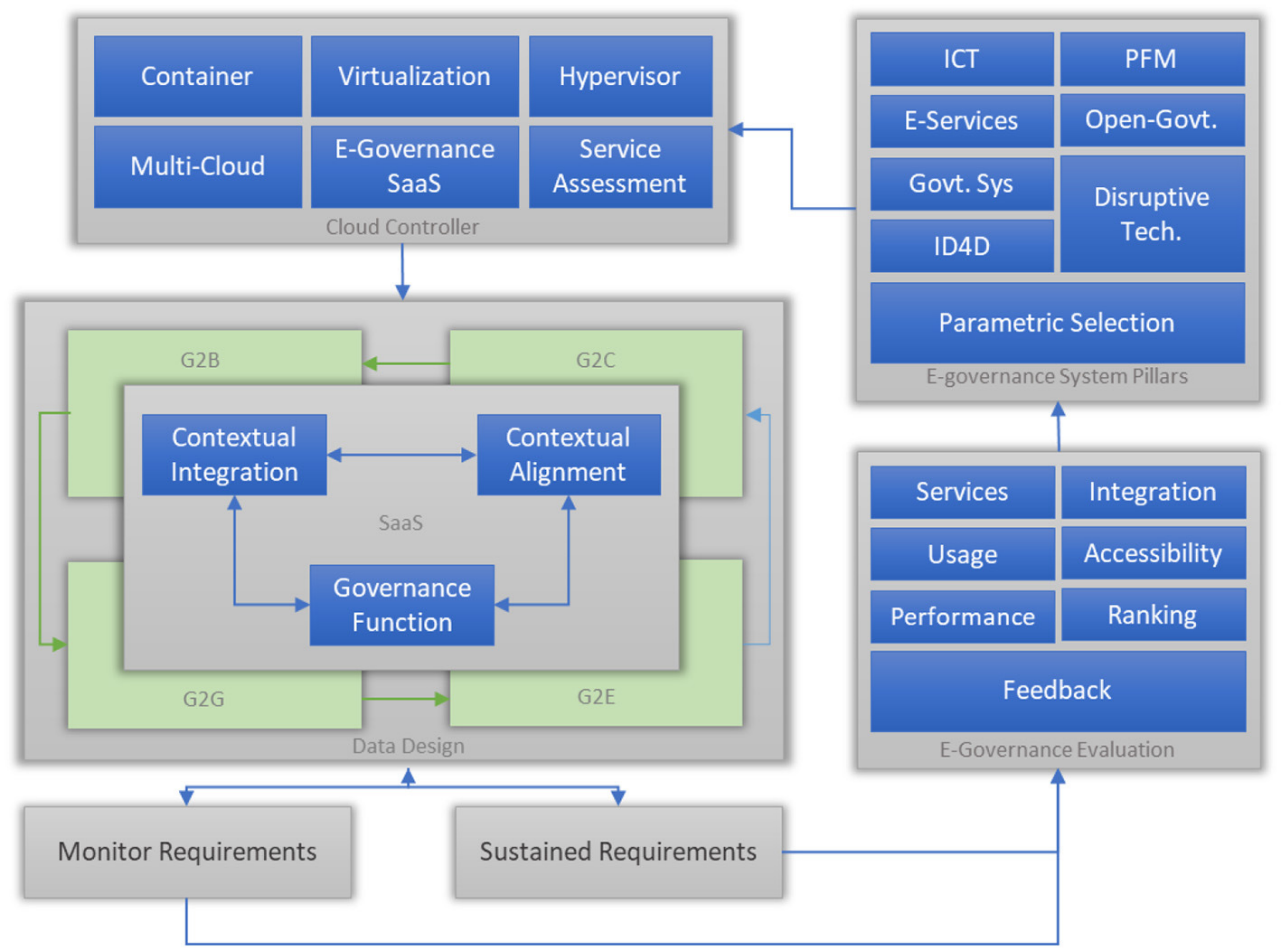
Conceptual description of the proposed solution.

The proposed system, MASC, has multiple components, as shown in Figure 2. These components depict functional entities and their associations with other entities. The diagram is developed based on a workflow structure to expose the functionality, flow, and linkages between multiple entities.

As the objective of MASC is to sustain a national-level system, this module is getting meta properties to maintain a balance and coherence among all entities to ensure smooth, long-term processes and procedures. This module contains multiple analytical features to evaluate the best parameters suitable for the governance system. The parameters will keep changing in preference due to the real-time data. The analytical regime contains key performance indicators, a preparation and implementation strategy, and a transition plan from moving one strategy to another. The sustain requirement module needs to be linked with the national database system or international governing databases to get data to initiate analysis compared with the current and localized data from the national real-time system/database. The parametric shuffling is required to keep the system updated, as shown in Figure 2.

The monitor requirement module gets the metadata of value properties to keep track of governance activities. Procedures developed and prioritized by the sustain requirement module are delivered by monitoring the requirement module to ensure deployment and are linked with the governance evaluation module. This module is linked with the monitoring and assessment engine and cloud data governance and preparation modules. The purpose of these connections is to provide maximum parameters for better monitoring and assessment. Furthermore, the monitoring and assessment engine has complete modulation for various types and categories of e-governance; the details are discussed in the following modular sections.

The e-governance pillar module takes care of the service provider side of the parameters, i.e., the government. It keeps track of services offered by the government, pre-requisites, workflow, and internal associations and relations of the services. In MASC, instead of developing a design that keeps a centralized approach, it is based on a component-based architecture, and components are arranged in a distributed manner; the distribution structure is based on the functionality of modules as in the cloud data governance office. The split is based on the two main actors of the e-governance system, i.e., the service provider, which is the government, and the service recipient, which in this case can be an individual citizen or an organization. The parameters of this module will keep changing based on the e-governance evaluation module. The key parameters will be selected based on the rank provided by the evaluation module.

The governance evaluation module is based on the parameters found through good global practices in e-governance design and deployment. In the same way, the Evolutionary Governance Theory (EGT) provides the governance structure and meaning as per international norms and standards. International agencies and different observation bodies are defining such parameters for the growth and sustenance of a national system deeply rooted in the society's social, financial, and human systems. All these factors play an implicit role in the development and deployment of any e-governance system.

The E-Governance Cloud Controller is responsible for overseeing the deployment, monitoring, and optimization of cloud-based applications, data storage, networking, and computing resources used in e-governance initiatives. Human societies, communities, cities, and countries are highly dynamic and keep improving and removing practices according to the needs and requirements of the majority. Similarly, an e-governance system should have dynamic features and a design that can help introduce new features and services and remove/hide or disconnect previous or undesired services and features.

This module is responsible for versioning of the implemented governance strategy and services. In case of any changes in the policy or services, it develops a new version and keeps the old version in the record. Considering the privacy and security of the data, all modules are independent in functioning, specifically cloud controllers. This module is responsible for provisioning virtual machines, storing data in containers, and making parametric functions into software as a service (SaaS) for accessibility through virtual machines or virtual instances created by the consumer. It also evaluates the services regarding performance, response time, and integration. In the cloud controller module, the proposed framework also provides the multi-cloud module that will cater to the multiple cloud service providers and their respective services in case any government wants to engage specific services or complete a platform from a cloud service provider.

MASC Scenario: The United Nations Department of Economic and Social Affairs (UNDESA) conducted the U.N. e-government survey in 2001^2^. UNDESA conducts a quarterly survey report evaluating the implementation status of all U.N. member states in e-government. The methodology used to conclude the analytical portion of the survey is built on a literature review and an analysis of data from the survey (Barrenechea and Jenkins, 2014).

The E-Government Development Index (EGDI) is used to measure the capacity and willingness of national administrations to use ICT to deliver government services, as shown in Table 1. The EGDI is focused on a systematic study of the online activity of all 193 members of the United Nations, which analyzes the national websites and how e-government policies and approaches are implemented in general and particular sectors to provide critical services (Kaur and Bala, 2015). EGDI is calculated on the average of three uniform scores.

- OSI (Online Service Index)
- TII (Telecommunication Infrastructure Index)
- HCI (Human Capital Index)

**Table 1:** E-government development index.

**EGDI**	**Value**
Very High	< 0.75
High	From 0.50 to 0.75
Medium	From 0.25 to 0.50
Low	>0.25

According to the 2018 survey, European nations lead to e-government growth globally. Asia and the Americas have nearly identical positions on middle and high e-government index rates. Several countries from Africa tend to fail to boost their e-government status. Out of 11, eight new countries that entered the very high-performing community in 2018 are from Europe and two from Asia. The overall progress of e-government development in Asia and the Americas is slow but noticeable, as shown in Table 2.

**Table 2:** EGDI based on region.

**Region**	**EGDI**	**EGDI group**
Europe	0.77	Very high
Americas	0.59	High
Asia	0.58	High
World	0.55	High
Oceania	0.46	Middle
Africa	0.34	Middle

Six countries from Asia registered an improvement in their online e-presence and provision of public services. Pakistan, Nepal, and Indonesia change from middle to high OSI. Cambodia, Timor Lester, and Tajikistan changed from low to middle OSI.

The E-Participation Index (EPI), Telecommunication Infrastructure Index (TII), and Human Capital Index (HCI) are based on the average online public consultations involving citizens in the decision-making process and the availability of online information. Denmark, Australia, and the Republic of Korea came out on top in a 2018 ranking of e-Government countries with EGDI values of 0.9150, 0.9053, and 0.9010. The rank of Pakistan is 148, with a value of 0.36. Table 3 compares Pakistan and its nearby countries.

**Table 3:** Comparison of countries in South East Asia.

**Country**	**EGDI rank**	**EGDI level**	**TIC**	**HCI**	**EG-DI**	**EPI rank**	**EPI**
China	65	High	0.47	0.71	0.68	15	0.96
Iran	86	High	0.45	0.74	0.61	112	0.53
Sri Lanka	94	High	0.31	0.75	0.58	86	0.63
India	96	High	0.20	0.55	0.57	15	0.96
Bangladesh	115	Middle	0.20	0.48	0.49	51	0.80
Pakistan	148	Middle	0.15	0.37	0.36	115	0.50
Afghanistan	177	Middle	0.11	0.36	0.26	145	0.32

The process of electronically delivering services to government clients, employees, and other government agencies is commonly known as e-government. Pakistan has also launched an e-government to bring about good governance and to facilitate the people of Pakistan. Most underdeveloped countries, such as Pakistan, can now introduce this form of government, which is only possible due to maturity and lower technology costs. The Pakistan Computer Bureau was made in 1971, while the directorate of e-governance was created in 2002. The Ministry of Information Technology was established in 2000. The primary need is to develop new IT colleges and open an information technology department in an existing institution.

Provide IT training to provincial and federal workers through the Computer Bureau to automate different government departments. Promote and strengthen the local information technology industry with the help of the Pakistan Software Export Board. Ministry of Information Technology has therefore used the Ministry of Planning Development and Special Initiatives (PSDP) budget of INR4.82 billion for these tasks. Between 2000 and 2004, a total of INR370 million was used for e-government projects.

Spammers, aiming to manipulate online reviews for the promotion or suppression of products, have become pervasive in the realm of online commerce. In response to this challenge, extensive research has been conducted to identify and combat review spammers. Many of these studies employ diverse features and subsequently create various classifiers in their efforts to detect and mitigate the impact of fraudulent reviews (Mell and Grance, 2011; Wu et al., 2020).

### 3.1 Case study for e-governance in Pakistan

The Electronic Government Directorate (EGD) was created in October 2002 under a federal cabinet resolution (Neto, 2011). The purpose of that unit is to employ various e-government-based initiatives to make professional guidance and suggestions accessible for conducting e-government programs. The re-engineering cycle has been carried out in all government departments for over 7 years. The National E-Government Council (NEGC) approved the 5-year governance policy in June 2005. The policy provides the infrastructure to every government department, provides citizens with e-services, and sets e-government project standards. The policy also brings awareness, attracts more citizens to use e-government services, and makes better understanding and delivery possible. Projects initiated by EGD are listed here in Table 4.

**Table 4:** National e-governance projects.

**Electoral rolls**	**All Ministries' and Divisions' websites**
E-complaint	Salary disbursement through ATMs
Crime reporting	Graphical information system
E-file tracking	E-archiving
Punjab intranet	Citizen online
Land record management information system	Motor vehicle registration
Prison management information system	Records digitization
Federal government web portal (www.pakistan.gov.pk)	Web archive management
Punjab province web portal (www.punjab.gov.pk)	Sindh government web portal (www.sindh.gov.pk)
National data warehouse	

Each of these projects is mainly focused on the public and common people. Some of the web portals mentioned above are online, as some are still under development but have already been approved by the Pakistan government for different IT companies. These projects aim to use the Internet to improve the quality of service for common people, providing a single entry point for both public and government information (Sarwar et al., 2023). The National Information Technology Board (NITB), sponsored by the Ministry of IT and Telecommunication, was formed on 11 August 2014 by merging the EGD Electronic government directorate and the Pakistan Computer Bureau (PCB).

Online platforms (Farokhi, 2015; Sarwar et al., 2021) for all agencies are accessible in developing nations with interactive and printable formats. Citizens can conveniently obtain information on all state notifications and programs. Pakistan, in general, and its province of Punjab have made considerable strides in digitizing their respective public sectors in recent years. Many government departments, including the private and business sectors, can be treated as e-government services. Out of many private and government departments (NITB), the National Information Technology Board and Punjab Information Technology Board (PITB) play an important role in developing e-government in Pakistan. NITB and PITB facilitate the successful implementation of e-governance programs by federal ministries to boost service and information provision, transparency, and efficiency. Pakistan is slowly progressing toward introducing e-governance to improve the availability and efficiency of knowledge and services offered to the public utilizing ICT in a fast and cost-effective way.

**Figure 3:**
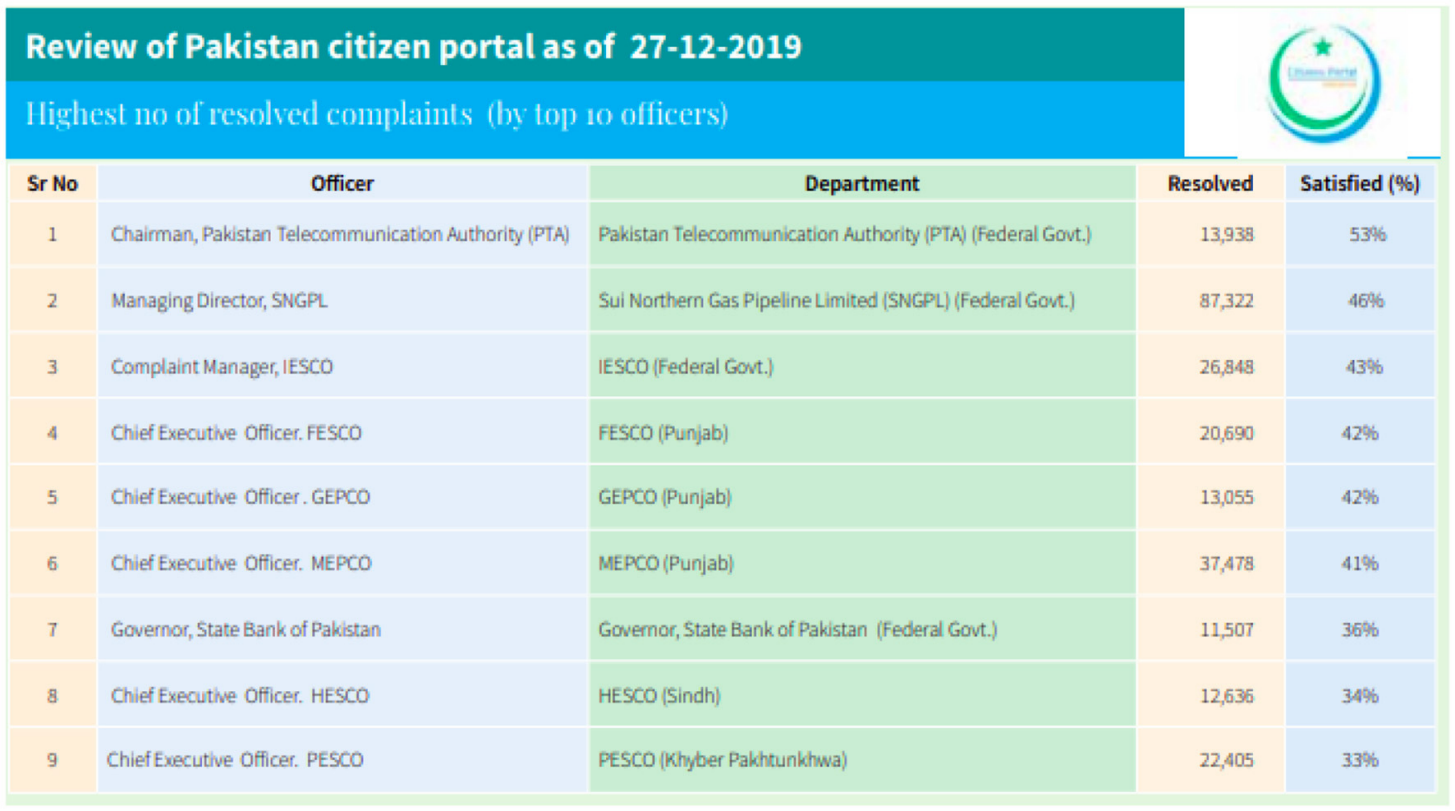
Pakistan citizen portal review report.

Pakistan Citizen Portal (PCP) is a mobile application launched on 28 October 2018. It has 1,000,000+ downloads and an average rating of 4.2 out of 5 on the Play Store. PCP is an integrated citizens' complaints resolution network that connects all federal and provincial government organizations. The app should act in Pakistan as the messenger of complaints to their relevant offices. People can submit complaints, and the concerned offices and departments must respond within a specific timeline. According to the PTA annual report, the 2019 PTA took the first position in resolving complaints, as shown in Figure 3.

The National Information Technology Board (NITB) was developed on 11 August 2014. The goal is to foster the wellbeing of Pakistani people across the globe by turning each ministry into a public entity with digital resources. Every ministry and province has its official website. Through these web portals, people can interact with government policies, tenders, offers, announcements, working projects, and basic information about the ministry and province. The government prefers creating websites that are reachable 24/7, which helps users access government information after visiting these websites, as shown in Table 5. Government workers are getting rid of face-to-face contact through government portals, and people conveniently send their applications. Pakistan Citizen Portal is one example where users can submit their complaints in every department through mobile applications.

**Table 5:** Applications working in Pakistan.

**Application name**	**Features**	**Average rating out of 5/number of download**	**Released on and developed by**
COVID-19	Self-assessment radio alert status	4.3/500,000+	27 Mar 2020 (NITB)
City Islamabad	Token text payment vehicle, registration, and details, city guide, domicile certification	3.7/10,000+	3 Mar 2020 (NITB)
Durust Dam	Old and new rates according to different perimeters	4.0/ 50,000+	8 Oct 2019 (NITB)
Baytee	App for Pakistan women, information about a police station, health services, updates on women's rights, laws, and new jobs	3.4/1,000+	14 Feb 2020 (NITB)
Epay Punjab	e-stamping, new vehicle registration, e-challan, property vehicle professional token tax, municipal of police complaints, domical license	3.8/50,000+	31 May 2019 (NITB)
eLearn App	Audio, digital practical books, quizzes	4.0/100,000+	31 May 2017 (PITB)
E-Khidmat Maraakaz	17 government services in one application	3.8/50,000+	14 Dec 2017 (PITB)
Pakistan online E-Services	Tracking link is provided near Pakistan electronic services	3.9/100,000+	3 Mar 2017 (Appscourt)
Rasta	Information about challan, car, license, and traffic advisory, schedule of e-driving test, e-license	3.9/100,000+	12 Jul 2017 PITB

Each application serves a specific purpose within the e-governance ecosystem, aiming to streamline and digitize traditional government services for the convenience of the public. The user ratings and number of downloads reflect the public's reception and adoption of these applications. The offering bodies, NITB and PITB, indicate a centralized effort to modernize and enhance accessibility to government services in Pakistan.

### 3.2 MACS core elements

The core elements of MACS are the same parameters that define the system's features and the governance's granular depth in accordance with the external stimulus. The e-governance system has the following main parameters:

a. ICT
b. Public Financial Management (PFM) System
c. Government System
d. E-Services
e. Open Government
f. Identification for Development (ID4D)
g. Disruptive Technologies

The aforementioned parameters are taken from good practices and World Bank digital global indicators and are mentioned in international simulations for digital forecasting and estimation of e-governance future. Various e-governance systems in developed countries consist of the aforementioned parameters, and the same is applicable to upcoming systems in developing countries. E-governance is moving around these points depending on the maturity of the system. The basic systems are exploring the ICT segment and utilizing the national infrastructure over the Internet to link various government departments, mostly focused on communication and better linkages between government segments. The root structure of global e-governance systems is based on ICT; further parameters are also identified using workflow theory for the validation purpose of MACS.

As the starting point of the e-governance system, the ICT segment workflow was evaluated using Microsoft Excel with time-series data ranging from 1999 to 2019. The following sub-structures are identified as shown in Table 6.

**Table 6:** ICT parameters.

**ICT parameters for e-governance**	
1	ICT sector/infrastructure investments
2	ICT for jobs
3	ICT industry and services
4	ICT literacy, skills development
5	ICT innovation and transformation
6	Science, technology, and innovation
7	ICT innovation policy
8	ICT innovation methodologies
9	Digital government
10	ICT strategy, policy, and regulation (incl. institutions)
11	ICT applications
12	ICT infrastructure
13	ICT methods and procedures
14	ICT for citizen engagement
15	IT security and audit
16	Cyber security
17	Telecommunications and broadband access
18	Telecommunications sector policy and regulation
19	Access and connectivity
20	Support for PPPs
21	Digital economy
22	Smart City

In global e-governance systems, these core parameters are treated for a sustainable e-governance system. MACS deals with these parameters with the help of the sustain module, as mentioned before. The aforementioned 22 parameters cover the basis of the e-services that can be developed on this broad foundation. At the same time, the other parameters contain their own sub-systems and parameters to grow the MACS structure further. The services any government develops with the help of the aforementioned parameters fall under the ICT structures and are easily deployable on cloud architecture. As mentioned earlier, a multi-cloud technique is better than a hybrid cloud for governments to maximize the benefits and optimum performance. Therefore, the 22 parameters can be deployed on a multi-cloud scenario using MACS structure, i.e., residing in a component approach using a sustain module.

#### 3.2.1 Public financial management

The most in-demand services for citizens and the government are related to public financials. This parameter is critically important as it is linked to the national economy, business structures, and individual financial capacity. The sub-parameters given below also show the relevance and coverage of the complete national economic cycle, as shown in Table 7.

**Table 7:** Public financial management parameters.

**Public financial management parameters for e-governance**	
1	Public financial management systems
2	Tax management systems
3	Customs management systems
4	e-procurement
5	Human resources management information system
6	Payroll management system
7	Asset management
8	Debt management
9	Treasury single account
10	Performance monitoring system
11	Financial management information system
12	Budget preparation
13	Program based budgeting
14	Public investment management
15	Treasury system
16	Accounting and reporting system
17	Audit management system
18	Aid management system
19	Natural resource management system

E-services, applications, and tools related to the aforementioned parameters are the common services required by individuals, SMEs, and large-scale organizations. Any e-governance system without comprehensive public financial management services and facilities cannot sustain and may not penetrate the masses to become a successful e-governance system.

Further segmentation of these sub-parameters is also possible in various developed countries, where many applications and e-services have been developed to provide a more comprehensive and autonomous structure for the public. It also helps the government make budgets and financial facilitation within the government departments as well as for the citizens of the country. Making large-scale projects and executing such projects within the stipulated timeframe is always a huge challenge, while connecting such a project management system with the aforementioned e-services provides a real-time management system with transparency and accuracy.

#### 3.2.2 Government system

Table 8 shows the functional sub-parameters suitable and practiced for government systems; again, one can see that there are many sub-parameters that are linked with public financial management and ICT parameters. These are the associative and relational parameters that connect these e-governance systems.

**Table 8:** Government system parameters.

**Government system parameters for e-governance**	
1	Sector-specific ICT applications
2	Payment systems
3	Education management information system
4	Health management information system
5	Health management information system
6	Pensions management information system
7	Land registration and cadastre system
8	Agriculture management information system
9	Statistical information systems
10	Justice information systems
11	Transport information systems
12	Geographic information systems
13	Management information systems
14	Digital health

#### 3.2.3 E-services

It is a generic fallacy when an e-governance system is considered a combination of e-services, although e-services are one of the very important parameters of the whole concept but not the whole system. The following are best practices and e-services that have been developed and implemented in e-governance systems, as shown in Table 9. The purpose of e-services is to provide a link between citizen and government applications and data repositories to utilize the services in a secure and transparent manner.

**Table 9:** E-services parameters.

**E-services parameters for e-governance**	
1	E-government portal and e-services
2	E-services portal
3	Informational e-services
4	Transactional e-services
5	Integrated e-services
6	Single sign on
7	Mobile e-service applications
8	Digital signature
9	Interoperability standards
10	Citizen engagement
11	Service centers

#### 3.2.4 Open government system

These sub-parameters are identified as the linked services for such applications that are technically on public cloud architecture, as the following table depicts the list of parameters related to national budgeting, contracts, and other such systems, which help make citizens and the government on one platform, as shown in Table 10.

**Table 10:** Open government system parameters.

**Open government system parameters for e-governance**	
1	Open government
2	System integration
3	Open government data
4	Open budget data
5	Open contracting
6	Open source software applications

#### 3.2.5 Identification for development

The purpose of identifying these parameters, mentioned in Table 11, is to have a sustainable e-governance system. The planned, expected, and strategic activities are a compulsory part of any governance system as they define the direction of growth and sustenance.

**Table 11:** ID4D parameters.

**ID4D parameters for e-governance**	
1	Identification for development
2	Civil registration
3	Identification systems
4	National ID
5	e-Passport
6	Unique business ID
7	Mobile ID applications
8	e-ID enabled services
9	Civil registration and vital statistics
10	Electoral systems
11	Functional registries
12	Business registry
13	Taxpayer ID
14	Civil service registry
15	Safety nets/beneficiaries
16	Health registries
17	Education registries
18	Transport registries
19	Asset registries
20	Natural resource licensing/registry

## 4 Results and discussion

### 4.1 Analysis 1995–2004

**Figure 4:**
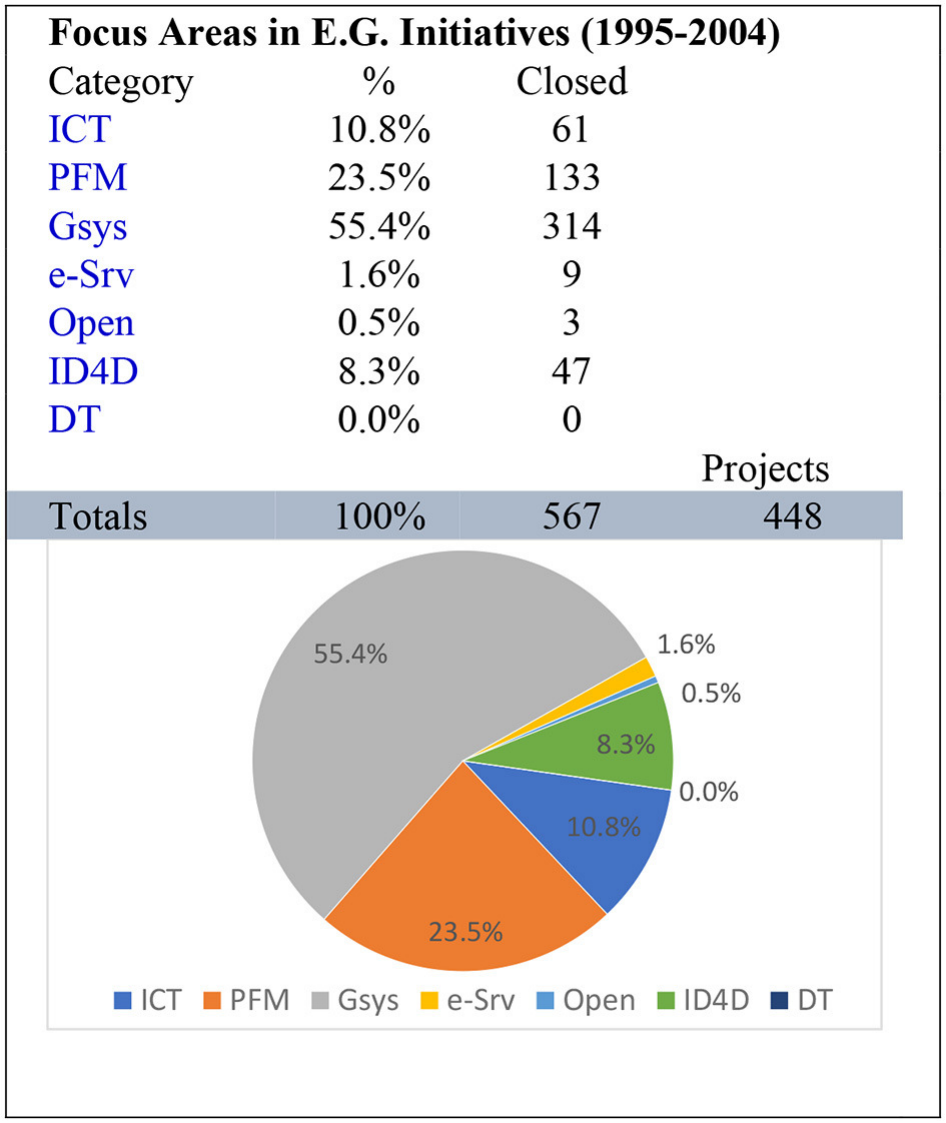
Analysis 1994–2004.

The dataset contains time-series data from 1995 to 2020 from 147 countries worldwide. The mentioned parameters indicate that between 1995 and 2004, government systems were the most concentrated area of interest in e-governance, representing 55.4% of all parameters. This suggests that our sustainable governance module is the valid unit containing all such parameters. Therefore, the government system is one critical monitoring parameter identified and analyzed for structuring any e-governance system, as shown in Figure 4.

### 4.2 Analysis 2005–2020

**Figure 5:**
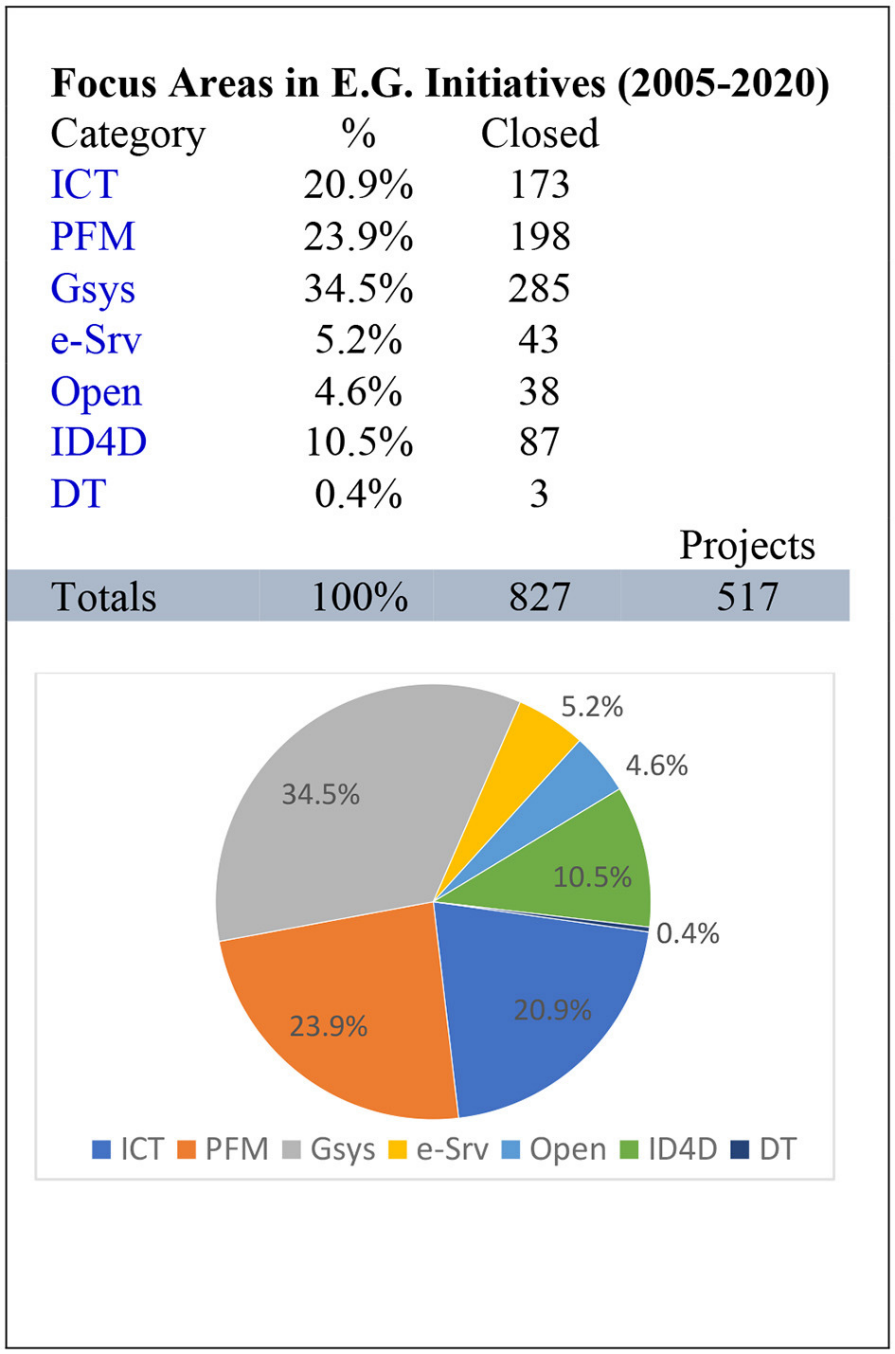
Analysis 2005–2020.

During the period 2005–2020, it is visible that the government systems were taking the lead, followed by the public financial management, and ICT is collecting more attention with a ratio of 20.9% as compared to the previous period of 10.8%, which means the double value, as shown in Figure 5. During the period 2005–2020, it is visible that the government systems were taking the lead, followed by the public financial management, and ICT is collecting more attention with a ratio of 20.9% compared to the previous period of 10.8%, which means the double value. Similarly, the monitoring parameters are increasing on ID4D from 8.3% to 10.5%, along with expanding e-services from 1.6% to 5.2%. as shown in Figure 5.

This is the rationale of the MASC government office and preparation modules. It is visible that from 1995 to 2020, the leading parameters gradually increased but were the same, i.e., government system, public financial management, and ICT, as explained in the core elements of the MASC. The secondary level parameters are gradually gaining value as the core parameters mature. With the passage of time and usage, the system has more users, more data, and more use cases to be structured as examples for the system's growth and further development of secondary parameters on solid, experienced grounds.

### 4.3 Analysis 2015–2020

**Figure 6:**
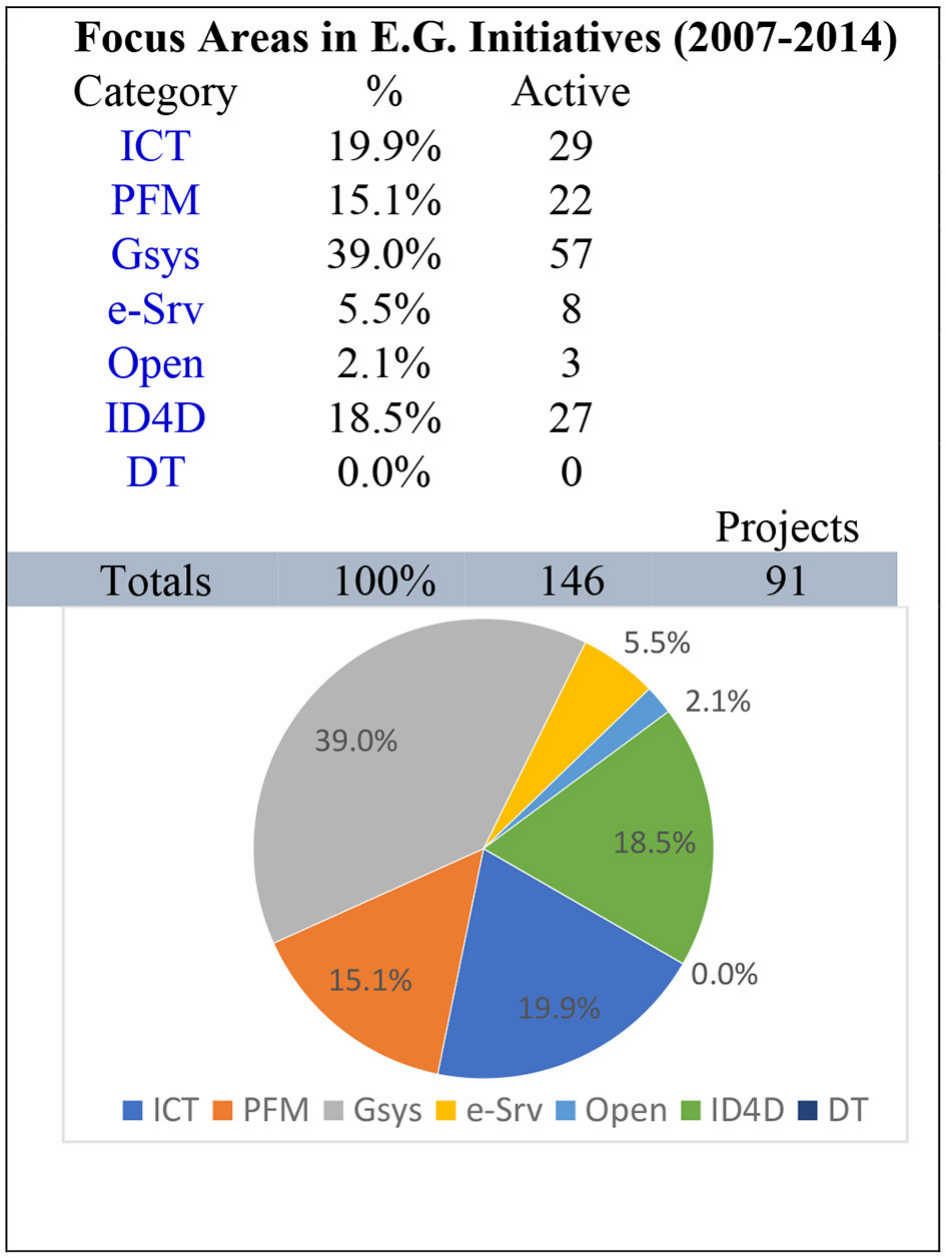
Analysis 2007–2014.

The next period is from 2007 to 2014, as shown in Figure 6. where the role of the government system is still larger than any other monitoring parameter, i.e., 39.0%, while more focus and development is visible in the ICT sector with a value of 19.9%, followed by a very close contender identification for development ID4D with a figure of 18.5%. This shows the system's maturity, as mentioned in the proposed framework, in that the monitoring engine is developing the coherence and balance between governance function, contextual alignment, and integration visible from the results. Core systems are gaining maturity and providing the basis for the secondary systems to root the tertiary-level applications. This also proves that the general perception about launching e-services may be a critical error without focusing on the real core factors exposed in these results.

These results show the conception of core elements of any e-governance system, and as the data is based on global good practices, it also depicts the prioritization in various cultures and economic situations. In other words, the same core elements are also required in developed countries (Sarwar et al., 2021).

### 4.4 Analysis 2015–2020

**Figure 7:**
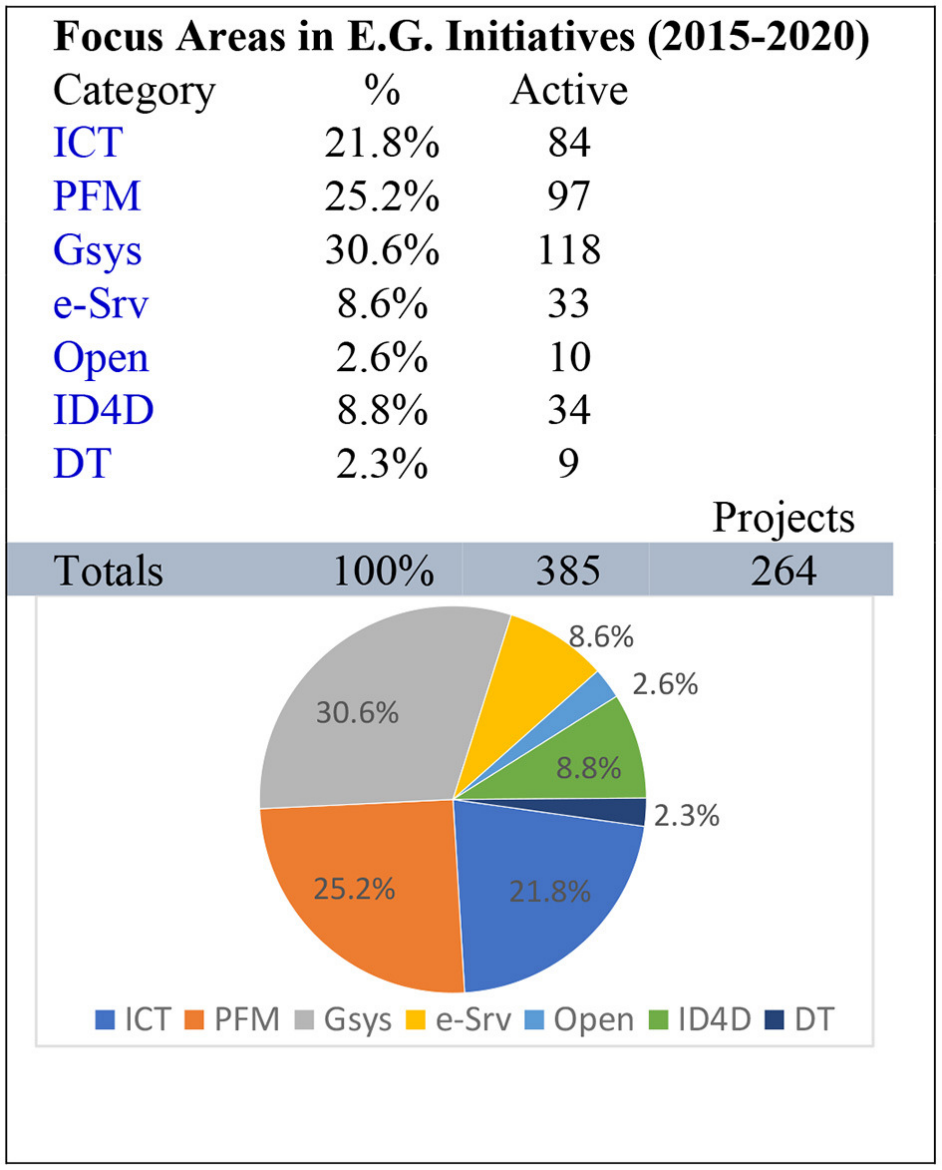
Analysis 2015–2020.

The final period in the time-series data covers from 2015 to 2020, as shown in Figure 7, the critical 5 years, as in 2019, when the world faced COVID-19, and that pandemic changed many predictions and forecasts in the global system theory. The interesting phenomenon is that globally, the countries with strong government systems worked better against the pandemic as the e-governance was already at a mature level; therefore, it was easy to offer preventive services against the pandemic using the same e-governance systems. The results table shows that the government system comprises 30.6% of the total, with public financial management increasing to 25.2%. Notably, even the pandemic issue did not alter the monitoring parameter ICT, which reached a value of 21.8%. The proposed framework MASC is also aligned with these results, i.e., integration and alignment of core elements with governance functions. Segregation of government offices and services separately is also showing promising results. The identified monitoring parameters and sub-parameters are strong value propositions for the e-governance systems and sustained activities in the future.

**Figure 8:**
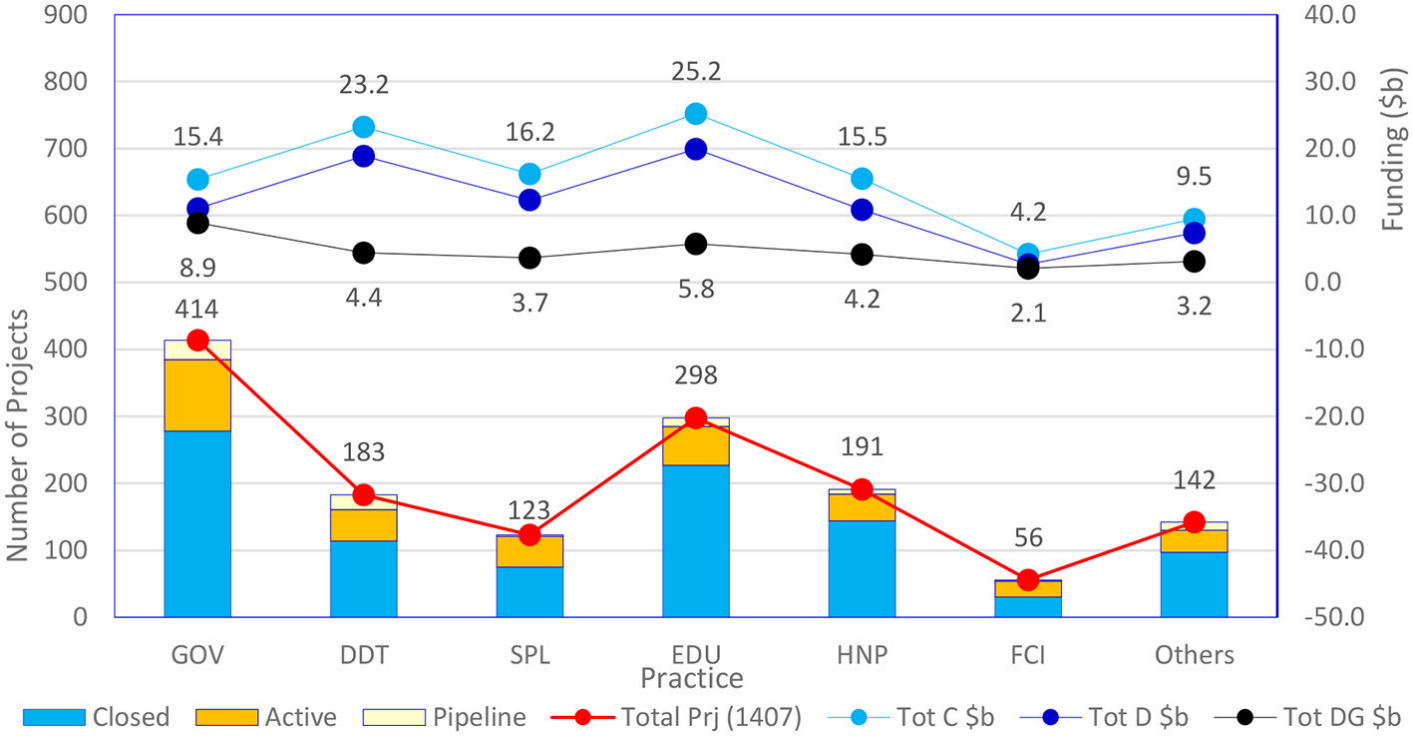
Sectoral distribution of e-governance initiatives.

Table 12 shows the sectoral prioritization and active, planned, and accomplished initiatives in e-governance. A total of 1,407 initiatives have been taken, worth 109.3b USD. The sectoral details show that most initiatives are related to government (GOV) and digital development (DDT), followed by social protection and job (SPL) and education (EDU). The other areas of global attention are health and nutrition (HNP) and financial competitiveness (FCI). The global development of e-governance, as shown in Figure 8 in terms of financials and prioritization, is available in this table and the following graph.

**Table 12:** Sectoral analysis of e-governance initiatives.

**Practice**	**Closed**	**Active**	**Pipeline**	**Total Prj (1,407)**	**Tot C $b**	**Tot D $b**	**Tot DG $b**
GOV	278	107	29	414	15.4	11.0	8.9
DDT	114	47	22	183	23.2	18.9	4.4
SPL	75	46	2	123	16.2	12.3	3.7
EDU	227	58	13	298	25.2	19.9	5.8
HNP	144	40	7	191	15.5	10.9	4.2
FCI	30	24	2	56	4.2	2.7	2.1
Others	97	33	12	142	9.5	7.4	3.2
	965	355	87	1,407	109.3	83.1	32.3

## 5 Discussion

Our meta-analysis reveals a recurring emphasis on citizen engagement, sustainability, and technology adoption within the realm of electronic governance. This meta-analysis, while showcasing the multifaceted dimensions of e-governance research, underscores the need for future studies that address the identified gaps, as shown in Table 13.

**Table 13:** Meta-analysis.

**Research focus**	**Methodology**	**Key findings**	**Contributions**
E-governance adoption in developing countries (Sharma and Thapliyal, 2011)	Quantitative survey	Increased citizen engagement; challenges in infrastructure	Insights for policymakers; highlights infrastructure gaps
Sustainability practices in e-governance (Seifert and Bonham, 2003)	Qualitative case study	Identified eco-friendly initiatives; barriers to adoption	Recommendations for sustainable e-governance strategies
Citizen participation in e-governance (Government of the United Kingdom, 2009)	Mixed methods approach	High citizen satisfaction with online services; areas for improvement	Insights for enhancing citizen engagement and service delivery

## 6 Conclusion

A framework, MASC, has been proposed containing components based on workflow mechanisms to explain the critical system entities and their relations and associations. Considering the existing cloud technology and skillsets, this framework is designed for cloud computing architecture. The component architecture has divided the MASC into two clear segments, i.e., the government/service provider segment that contains the functions and features to accommodate policies, services, and intra-government relations and associations. The other part of the MASC is related to developing the integration and alignment of context with governance functions. That is an important segment because, due to this contextual functionality, it is possible to identify future parameters using machine learning or other agency techniques. In other words, it will lead to the development of a complete taxonomy of e-governance in the future.

Time series analysis is applied to the period from 1999 to 2020 to identify the patterns in digital domains. The outcome is seven core elements or the core monitoring parameters for the e-governance system, and further analysis has provided 102 sub-parameters in 07 core elements. Furthermore, six top sectors have been analyzed to examine the core and primary elements. The results are in favor of the proposed framework MASC; it also exposes many interesting patterns to provide denial to different general perceptions related to e-governance and highlights the true actors that are playing a vital role in the development, sustenance, and growth of e-governance systems using cloud computing architecture in a global perspective.

## Author contributions

QA: Conceptualization, Data curation, Validation, Visualization, Writing – original draft. TAly: Conceptualization, Methodology, Writing – original draft. TAlg: Formal analysis, Investigation, Methodology, Writing – review & editing. AA: Conceptualization, Data curation, Formal analysis, Writing – review & editing. SA: Formal analysis, Investigation, Methodology, Writing – review & editing. MN: Writing – review & editing. NT: Investigation, Methodology, Validation, Writing – original draft.

## Footnotes

^1^Available online at: http://www.informationweek.com/shared/printableArticle.jhtml?articleID=217500403.

^2^Available online at: http://www.futuregov.net/articles/2009/may/25/thailand-plansprivate-cloud-e-gov.
